# Improve protective efficacy of a TB DNA-HSP65 vaccine by BCG priming

**DOI:** 10.1186/1479-0556-5-7

**Published:** 2007-08-22

**Authors:** Eduardo DC Gonçalves, Vânia Luiza D Bonato, Denise M da Fonseca, Edson G Soares, Izaíra T Brandão, Ana Paula M Soares, Célio L Silva

**Affiliations:** 1 Farmacore Biotecnologia Ltda, Rua dos Técnicos s/n, Campus da USP, Ribeirão Preto, SP, Brasil; 2 Center for Tuberculosis Research, Department of Biochemistry and Immunology, School of Medicine of Ribeirão Preto, University of São Paulo, Brazil; 3 Department of Pathology, School of Medicine of Ribeirão Preto, University of São Paulo, Brazil

## Abstract

Vaccines are considered by many to be one of the most successful medical interventions against infectious diseases. But many significant obstacles remain, such as optimizing DNA vaccines for use in humans or large animals. The amount of doses, route and easiness of administration are also important points to consider in the design of new DNA vaccines. Heterologous prime-boost regimens probably represent the best hope for an improved DNA vaccine strategy. In this study, we have shown that heterologous prime-boost vaccination against tuberculosis (TB) using intranasal BCG priming/DNA-HSP65 boosting (BCGin/DNA) provided significantly greater protection than that afforded by a single subcutaneous or intranasal dose of BCG. In addition, BCGin/DNA immunization was also more efficient in controlling bacterial loads than were the other prime-boost schedules evaluated or three doses of DNA-HSP65 as a naked DNA. The single dose of DNA-HSP65 booster enhanced the immunogenicity of a single subcutaneous BCG vaccination, as evidenced by the significantly higher serum levels of anti-Hsp65 IgG2a Th1-induced antibodies, as well as by the significantly greater production of IFN-γ by antigen-specific spleen cells. The BCG prime/DNA-HSP65 booster was also associated with better preservation of lung parenchyma.

The improvement of the protective effect of BCG vaccine mediated by a DNA-HSP65 booster suggests that our strategy may hold promise as a safe and effective vaccine against TB.

## Background

Tuberculosis (TB) remains a leading cause of infectious disease mortality worldwide, accounting for nearly 2 million deaths annually. Despite the availability of effective anti-TB therapy, the world's case burden of TB continues to climb, in part owing to the concurrent acquired immune deficiency syndrome pandemic. The widespread use of the current TB vaccine, *M. bovis *bacillus Calmette-Guérin (BCG), has failed to curtail the TB epidemic. Therefore, TB eradication will require the development of an improved vaccine, which, in turn, will require application of state-of-the-art vaccine technology and new strategies.

A new vaccine against TB would need to induce protection superior to that elicited by the BCG vaccine and to permit administration to healthy individuals, infected individuals and perhaps even individuals presenting the active form of the disease. Thus, various strategies have been employed for the development and evaluation of new TB vaccines. Recombinant BCG strains, DNA-based vaccines, live attenuated *Mycobacterium tuberculosis *vaccines and subunit vaccines formulated with novel adjuvants have shown promise in preclinical animal models [1]. The ability of DNA vaccines to elicit Th1-biased CD4^+ ^responses and strong cytotoxic T lymphocyte responses make them particularly attractive as weapons against *M. tuberculosis *infection.

Experimental data collected by our group over the last few years have shown that a DNA vaccine encoding the *M. leprae *65-kDa heat shock protein (DNA-HSP65) has prophylactic and therapeutic effects in a murine model of TB [2-5]. The prophylactic effect initially obtained from this vaccine was equal to that elicited by BCG vaccine [3,6]. However, we would like to optimize this DNA vaccine for use in humans, and the prime-boost strategy seems a very promising option.

Heterologous prime-boost strategy has shown promise in various models of pathogenic infections [7]. The results have been highly encouraging both in augmenting and modulating vaccine-induced immunity. This strategy is based on the combination of live attenuated viruses or BCG with DNA vaccines or recombinant proteins [8]. In experimental models of TB, the ability of prime-boost strategy to complement the protection provided by BCG vaccination has been assayed [9]. Such studies have shown that DNA-prime that codifying *M. tuberculosis *genes (Apa, HSP65 and HSP70), BCG-booster induced a higher level of protection than BCG alone [10]. However, boosting the BCG vaccine with a recombinant modified vaccinia virus Ankara (MVA) expressing *M. tuberculosis *85A antigen also induced higher levels of antigen-specific CD4^+ ^and CD8^+ ^T cells and greater protection against aerosol challenge [11]. Others have demonstrated that BCG-prime DNA-Rv3407 (*M. tuberculosis *10 kDa protein)-booster induced a greater protection against TB than BCG alone [12]. In the present study, we investigated the influence that the order and route of BCG vaccination in combination with DNA-HSP65 vaccine has on the induction of protective immunity against TB.

## Methods

### Mice

SPF female BALB/c mice, 6–8 weeks old, were purchased from the University of São Paulo – FMRP. All mice were kept under specific pathogen-free conditions in a BSL 3 facility. All animal studies were conducted in accordance with the Institutional Animal Care and Ethics Rules of University of São Paulo – Brazil.

### Bacteria

The *M. tuberculosis *H37Rv (n° 27294; ATCC, Rockville, MD, USA) and *M. bovis *BCG (Pasteur strain) were grown in an incubator for 7 days at 37°C in 7H9 Middlebrook broth (Difco, USA) enriched with 0.2% (v/v) glycerol and 10% (v/v) OADC (Difco, USA) and was prepared as described [5].

### Plasmid construction

The DNA vaccine pVAX-hsp65 (DNA-HSP65) was derived from the pVAX vector (Invitrogen, Carlsbad, CA, USA) and was constructed as described [13]. Endotoxin levels were measured using the Limulus amebocyte lysate kit – QCL-1000 (BioWhittaker, Walkersville, MD, USA). Endotoxin levels for plasmid used in this study were ≤ 0.1 endotoxin units/μg of DNA.

### Immunization and challenge infection

Groups of mice were separated by immunization schedule as shown in Table 1. For DNA vaccination, a single 50-μg dose of DNA-hsp65 in 50 μL of saline plus 50% sucrose was injected into each quadriceps muscle 3 times in a 15 day-intervals by using insulin syringe with an ultra-fine II short needle (Becton and Dickson, Franklin Lakes, NJ – USA). For intranasal (i.n.) delivery of BCG, animal groups were lightly anesthetized with tribromoethanol 2,5% (Across Organics) and 10^5 ^bacilli in 30 μl of PBS/mouse was administered dropwise to external nostrils of the mice (15 μl per nostril) with a fine pipette tip. For subcutaneous (s.c.) delivery, animals received 10^5 ^bacilli in 100 μl of PBS/mouse. At 15 or 60 days after the last immunization, mice were challenged through instillation of bacterial solution (10^5 ^bacteria/animal) by intratracheal route according to harmonization procedures of animals. For each route of immunization and challenge an equal quantity of PBS was administered to the controls.

**Table 1:** Heterologous prime-boost regimen combinations

**GROUP**	**PRIME**	**BOOSTER**^a^
**PBS**	PBS	PBS
**BCGsc**	Subcutaneous^b ^BCG	-
**BCGin**	Intranasal^c ^BCG	-
**BCGin/DNA**	Intranasal BCG	Intramuscular^d ^DNA-hsp65
**BCGsc/DNA**	Subcutaneous BCG	Intramuscular DNA-hsp65
**DNA-hsp65**	3 doses of intramuscular^d^	DNA-hsp65 – 15 days of interval

^a^Interval between prime and booster: 15 days

^b^Subcutaneous route: 10^5 ^bacteria in 30 microliters

^c^Intranasal route: 10^5 ^bacteria in 100 microliters

^d^Intramuscular route: 100 micrograms in 100 microliters/dose, 3 doses at 15-day intervals

### Blood collection and antibody evaluation

Prior to the first immunization (pre-immune serum) and 15 days after the last immunization, individual serum samples were colleted by retro-orbital sinus puncture. Antibody levels in samples were measured by enzyme-linked immunosorbent assay (ELISA) described [13].

### Recombinant *M. leprae *hsp65

Clone pIL161, containing the DNA coding for the *M. leprae *HSP65, was transformed into electrocompetent DH5α Escherichia coli cells. Briefly, DH5α *E. coli *cells containing pIL161 were grown in the presence of ampicillin to an OD600 of 0.6. The expression of the recombinant protein was induced by the addition of IPTG (isopropril-thi-B-D-galactosídeo) 0.5 mM. The induced culture was incubated for another 4 h at 30°C and was harvested by centrifugation (5000 g, 5 min, 4°C), then the pellet was lysed by sonication at 60 Hz with two cycles of 60 s (Tomy-Seiko, Japan). After washed with 10 mL of CE buffer, the pellet was resuspended in 5 mL of UPE buffer and the suspension was gently shaken at room temperature for 15 min. The insoluble material was washed by centrifugation at 10000 g for 20 min, a 3.6 M ammonium sulfate stock solution was added followed by incubation on ice for 30 min. This fraction was dissolved in 50 mM phosphate buffer to produce the crude fraction. The recombinant *M. leprae *Hsp65 was first fractionated on a FPLC-GP-250 Plus system (MonoQ HR 5/5, Pharmacia Biotech) using 50 mM phosphate buffer and eluted with a 20–600 mM NaCl gradient under a flow rate of 1 mL/min. Subsequently, the protein solution (100 μg) was resolved on a HPLC system (Shimadzu Class VP) and recombinant *M. leprae *Hsp65 was collected and the homogeneity of the recombinant *M. leprae *Hsp65 preparations was analyzed by polyacrylamide gel electrophoresis. Protein concentrations and endotoxin levels were determined as previously described [5,14].

### Cytokine detection

The levels of IFN-γ, interleukin (IL)-12, IL-10, TNF-α, IL-4 and IL-5 in the spleen cell supernatants and in lung homogenates from immunized mice were measured by ELISA as previously described [5]. The following capture antibody anti-mouse IFN-γ, IL-12, IL-10, TNF-α, IL-4 or IL-5 (R46A2, 15.6, JES5-2A4, mIL4R-M2 and TRFK5 clones, respectively; Pharmingen) were used. Cytokine-antibody complexes were detected by the addition of biotin anti-mouse IFN-γ, IL-12, IL-10, TNF-α, IL-4 or IL-5 (XMG1.2, C17.8, SXC-1, B11-3 and TRFK4 clones, respectively; PharMingen). Detection limits were 40 pg/mL (for IFN-γ and 10 pg/mL (for IL-12 and IL-10, TNF-α, IL-4 and IL-5).

### Elispot Assay

The ELISPOT method was used to detect IFN-γ secretion by spleen cells from immunized mice. In brief, ELISPOT plates (BD Biosciences) were coated with capture IFN-γ antibody overnight at 4°C. After washed and blocked with complete medium, the plates were incubated for 2 h at room temperature. The spleen was removed from each mouse aseptically. Red blood cells were removed from the spleen cells preparations using red blood cell lysis buffer (NH_4_Cl 0,16 M/Tris 0,17 M/pH 7,65). Cells were placed in RPMI-C 1640 medium (R-6504 – Sigma, St. Louis, USA) supplemented with 100 U/mL penicillin, 100 μg/mL streptomycin, and 10% of fetal bovine serum (all from Gibco-BRL). The cells were incubated (2 × 10^6 ^cells/well) for 48 h at 37°C with 5% CO_2_, with medium, concanavalin-A (20 μg/well) or recombinant Hsp65 (10 μg/well) and then were discarded. Plates were washed with de-ionized water and PBS/Tween 20. Secondary biotinylated antibody was added for 2 h and incubated at room temperature, followed by washing with PBS/Tween 20. Streptavidin-alkaline phosphatase was added to the plates for 1 h, followed by washing and by the development of a colour reaction using the AEC substrate reagent kit (BD Biosciences). The reaction was stopped by rinsing the plate with running water. The spots were enumerated using an ELISPOT reader (Biosys – Germany).

### Protection assay

Thirty days after challenge, aliquots of lungs harvested from infected, sham-immunized mice and from immunized, infected mice were incubated in digestion solution as described [5]. Serial 10-fold dilutions were plated on supplemented 7H11 agar media (Difco, USA). Colonies were counted after 28 days of incubation at 37°C with 5% CO_2_, and the results were expressed as CFU (g/lung).

### Preparation of lung cells

Lungs were washed with sterile PBS and were placed in Petri dishes containing incomplete RPMI-1640 (R-6504 – Sigma, St. Louis, USA). Then, they were fragmented and transferred to conical tubes containing 0.5 μg/mL of Liberase Blendzyme 2 (Roche, Indianapolis, IN, USA) in incomplete RPMI-1640. Samples were processed as previously described [5].

### Fluorescence-activated cell sorter analysis

To evaluate T cell subsets, effector function and memory markers, the following mAbs and their respective isotype controls were used: anti-CD62L (clone MEL-14), anti-CD4 (clones H129.19 and RM4-5), anti-CD8 (clone 53-6.7), anti-CD44 (clone Ly-24); rat-IgG2a-fluorescein isothiocyanate, rat-IgG2a-phycoerythrin and rat-IgG2a-peridinin chlorophyll protein. All mAbs were purchased from Pharmingen and used according to the manufacturer instructions. Lymphocytes were analyzed by flow cytometry using the CellQuest software FACSort (Becton Dickinson, San Jose, CA). Ten thousand events per sample were collected, and three-color fluorescence-activated cell sorter analysis was performed. Expression of CD62L^lo ^and CD44^hi ^was performed by dot plot in CD4^+ ^or CD8^+ ^gated lymphocyte populations.

### Histology

Lung samples were fixed in 10% buffered formalin. Five-micrometer sections were stained with hematoxylin-eosin and the granulomatous lesions were analyzed by light microscopy (Leica, Germany).

### Statistical analysis

All data were analyzed individually and the values were expressed as mean ± SEM. When the values indicated the presence of a significant difference by analysis of variance (ANOVA), a Tukey-Kramer multiple comparisons test was used. Values of *P *<0.05 were considered significant.

## Results

### DNA-HSP65 boosting of BCGin provides greater protection than other immunization strategies

**Figure 1:**
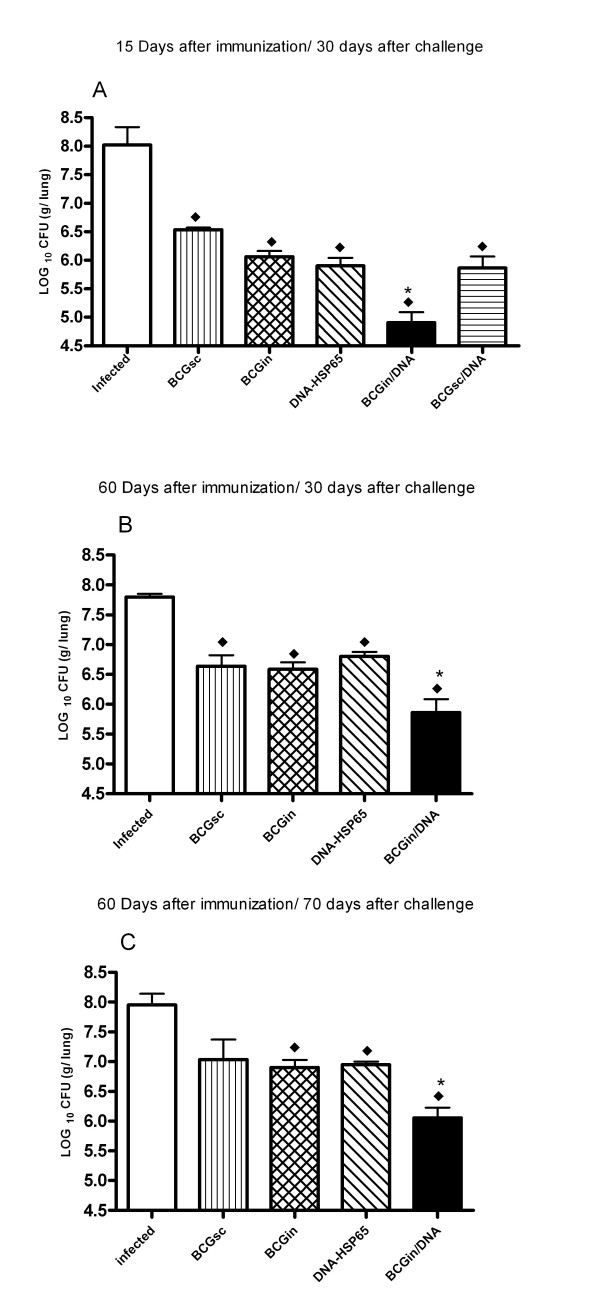
Prime-boost strategy-induced protection against *M. tuberculosis *challenge. Groups of 7 BALB/c mice were immunized as shown in Table 1. Control mice received PBS prior to infection (infected group). (A) Fifteen days after the last immunization, the mice were challenged with *M. tuberculosis*. Thirty days later, the number of live bacteria in the lungs was determined and expressed as CFU/lung. (B, C) Sixty days after the last immunization, the mice were challenged and 30 (B) and 70 (C) days later, the number of CFU was determined. Bars represent the mean ± standard deviation. (A) ◆ all immunized-infected mice vs infected mice. * BCGin/DNA vs BCGsc, BCGin, DNA-HSP65 and BCGsc/DNA. (B) ◆ BCGin/DNA, DNA-HSP65 and BCGin vs infected mice. * BCGin/DNA vs BCGsc, BCGin and DNA-HSP65. (C) ◆ BCGin, DNA-HSP65 and BCGin/DNA vs infected mice. * BCGin/DNA vs BCGsc and DNA-HSP65. p < 0.05 was considered significant. Data are representative of two experiments.

Initially, we tested the ability of different combinations of prime-boost strategies to induce protection against *M. tuberculosis *and compared the results with those obtained using classical BCG vaccination or naked DNA-HSP65 immunization through the detection of the number of colony-forming units (CFU). Significant protection against experimental TB was achieved in all immunized, infected mice using the various prime-boost strategies (Table 1) or three DNA-HSP65 homologous immunizations or a single BCG dose (BCGin or BCGsc) (Fig. 1A). However, BCGin/DNA immunized, infected mice presented a reduction of 3,1 LOG_10 _in the lung, a significantly greater degree in relation to non-immunized, infected group (Fig. 1A). The other immunized, infected mice also presented a significant reduction, as follows: BCGsc (1,49 LOG_10_), BCGin (1,94 LOG_10_), DNA-hsp65 (2,1 LOG_10_), BCGsc/DNA (2,14 LOG_10_). DNA prime/BCG booster also induced significant protection in relation to non-immunized, infected group (data not shown). Additionally, when compared with BCGsc (1,63 LOG_10_), BCGin (1,16 LOG_10_) and DNA-HSP65 (1,0 LOG_10_), the BCGin/DNA group presented a significant reduction of bacterial load (Fig. 1A). In another set of experiments, the challenge was performed 60 days after the last immunization and 30 or 70 days post-infection the CFU burden recovered from lungs was determined (Fig. 1B and 1C), respectively. We verified 30 days post-infection that all immunized, infected groups presented significant reduction of CFU in the lung when compared with infected group (Fig. 1B). Moreover, only BCGin/DNA group presented significant reduction of CFU compared with the other groups: BCGin (0,62 LOG_10_), BCGsc (0,67 LOG_10_) and DNA-HSP65 (0,84 LOG_10_) (Fig. 1B). When the bacterial burden was determined by CFU analysis 70 days post-infection, a significant reduction of burden was observed in BCGin, DNA-HSP65 and BCGin/DNA groups (1,22 LOG_10_, 1,05 LOG_10 _and 1,75 LOG_10_, respectively) compared with infected group (Fig. 1C). In addition, BCGin/DNA group also presented a significant reduction in the number of CFU when compared with BCGin (0,53 LOG_10_) and DNA-HSP65 (0,74 LOG_10_) groups (Fig. 1C).

### BCGin/DNA induces enhanced humoral immune response

**Figure 2:**
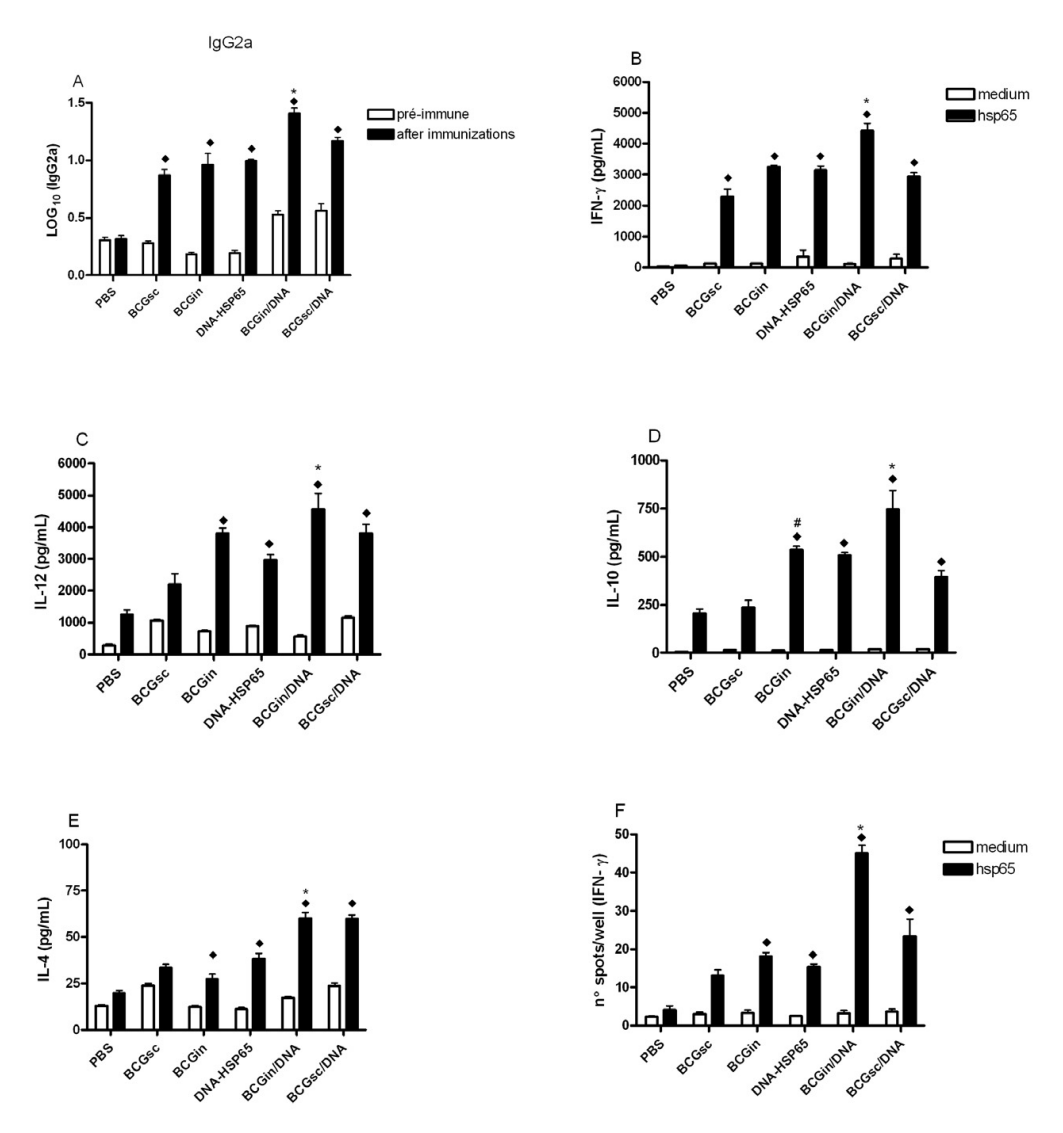
Specific immune response detected in mice immunized or sham-immunized as described in Table 1. (A) IgG2a specific antibodies detected in serum collected before the first immunization (pre-immune) and 15 days after the last immunization. Detection of cytokines in supernatants of spleen cell cultures and IFN-gamma-producing cells (spleen cells) detected by ELISPOT. Groups of 7 mice were sacrificed 15 days after the last immunization, and the spleen cells were cultured in medium with or without rhsp65 (10 micrograms/well). (B) IFN-gamma, (C) IL-12, (D) IL-10, (E) IL-4 and (F) IFN-gamma spots. Bars represent the mean ± standard deviation. (A) ◆ all immunized-mice vs Infected. * BCGin/DNA vs BCGin, BCGsc, DNA-HSP65 and BCGsc/DNA. (B) ◆ all immunized-mice vs Infected. * BCGin/DNA vs BCGsc, BCGin, DNA-HSP65 and BCGsc/DNA. (C) ◆ BCGin, DNA-HSP65, BCGin/DNA and BCGsc/DNA vs Infected. * BCGin/DNA vs BCGsc, DNA-HSP65 and BCGsc/DNA. (D) ◆ BCGin, DNA-HSP65 and BCGin/DNA vs Infected. * BCGin/DNA vs BCGsc, DNA-HSP65 and BCGsc/DNA. # BCGin vs BCGsc. (E) ◆ BCGsc, DNA-HSP65, BCGin/DNA and BCGsc/DNA vs Infected. * BCGin/DNA vs BCGsc, BCGin and DNA-HSP65. (F) ◆ BCGin, DNA-HSP65, BCGin/DNA and BCGsc/DNA vs Infected.* BCGin/DNA vs BCGsc, BCGin, DNA-HSP65 and BCGsc/DNA. p < 0.05 was considered significant. Data are representative of two experiments.

In order to evaluate the humoral immune response in serum from all immunized mice and control group (PBS-injected group) the serum was collected before (pre-immune) and 15 days after the last immunization. We verified that all immunized mice presented significant levels of anti-Hsp65 IgG2a after 15 days of the last immunization (Fig. 2A). Moreover, BCGin/DNA immunized-mice produced significant levels of IgG2a, (1,41 LOG_10_) in relation to mice immunized with a single dose of BCGin (1,06 LOG_10_) or BCGsc (0,86 LOG_10_) and a homologous DNA-HSP65 immunization (1,0 LOG_10_) (Fig. 2A). No significant differences were found among specific IgG1 antibody levels collected post-immunizations in relation to PBS group (data not shown).

### BCGin/DNA stimulates a Th1 immune response

To evaluate the specific cytokine production from spleen cell cultures of non-immunized and immunized-mice 15 days after the last immunization, the ELISA assay was performed. After specific stimulation with rHsp65, the spleen cells of all immunized-mice, BCGsc (2275 ± 807 pg/mL), BCGin (3256 ± 120 pg/mL), DNA-HSP65 (3139 ± 383 pg/mL), BCGsc/DNA (2931 ± 430 pg/mL), produced significant levels of IFN-γ in relation to PBS group (Fig. 2B). However, the spleen cells of BCGin/DNA immunized-mice produced significantly higher levels of detectable IFN-γ (4411 ± 799 pg/mL) compared with the levels provided by other immunized mice (Fig. 2B). A similar pattern of cytokine production was observed in relation to IL-12, with the exception of BCGsc group that did not produce significant levels of IL-12 in relation to PBS group (Fig. 2C). The other groups of immunized-mice produced different levels of IL-12: BCGin/DNA (5025 ± 747 pg/mL), BCGsc (2208 ± 1055 pg/mL), BCGin (3803 ± 385 pg/mL), DNA-HSP65 (2962 ± 474 pg/mL) and BCGsc/DNA (3806 ± 942 pg/mL) (Fig. 2C). On top of that, the levels of IL-12 produced by BCGin/DNA group were significantly higher than those produced by DNA-HSP65 group (Fig. 2C). In addition to identifying the cytokines IFN-γ and IL-12, which are associated with the Th1 pattern, we found that the BCGin/DNA immunization schedule stimulated significantly higher IL-10 production (627 ± 174 pg/mL) compared with that provided by BCGsc (237 ± 110 pg/mL) and BCGsc/DNA (393 ± 102 pg/mL) groups (Fig. 2D). Similar levels of IL-10 were produced by BCGin/DNA, BCGin and DNA-HSP65 groups. Besides, we verified that DNA-HSP65 (38 ± 7 pg/mL), BCGin/DNA (59 ± 10 pg/mL) and BCGsc/DNA (59 ± 6 pg/mL) immunized-mice also produced significant levels of IL-4 compared with PBS group (Fig. 2E). In relation to ELISPOT assay, significant number of IFN-γ producing cells was mainly observed in BCGin/DNA mice (45 spots) in comparison with the number detected in PBS group and in the other immunized groups: BCGin, DNA-HSP65, BCGsc/DNA and BCGin/DNA (Fig. 2F).

### Maintenance of Th1-type response after challenge with *M. tuberculosis*

**Figure 3:**
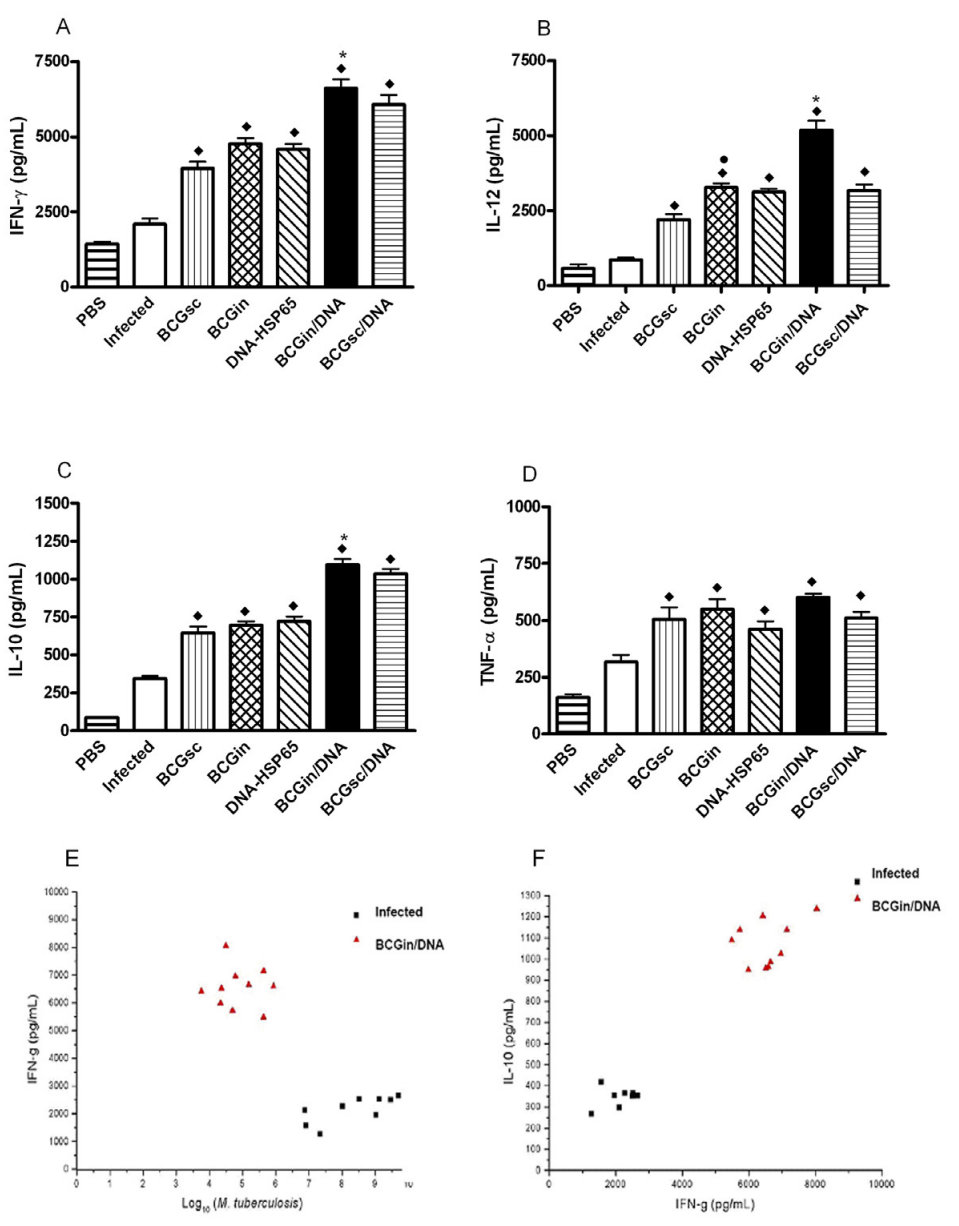
IFN-gamma, IL-12, IL-10 and TNF-alpha in lung homogenates from immunized, infected mice and infected mice after 30 days of the infection. (A) IFN-gamma; (B) IL-12; (C) IL-10 and (D) TNF-alpha. (E) Correlation between CFU numbers and IFN-gamma production (F) Correlation between IFN-gamma and IL-10 production. Groups of 7 mice were immunized according table I and 15 days after the last immunization, they were challenged with H37Rv. After 30 days of infection, the lungs were removed and the cytokine production in lungs homogenates was analyzed. Bars represent the mean ± standard deviation. (A) ◆ BCGsc, BCGin, DNA-HSP65, BCGin/DNA and BCGsc/DNA vs Infected mice. * BCGin/DNA vs BCGsc, BCGin, and DNA-HSP65. (B) ◆ All immunized-infected mice vs Infected mice. * BCGin/DNA vs BCGsc, BCGin, DNA-HSP65 and BCGsc/DNA. ● BCGin vs BCGsc. (C) ◆ All immunized-infected mice vs Infected mice. * BCGin/DNA vs BCGsc, BCGin, DNA-HSP65. (D) ◆ All immunized-infected mice vs Infected mice. p < 0.05 was considered significant. Data are representative of two experiments.

In order to evaluate the profile of immune response after mycobacterial challenge, the cytokine production in lung homogenates was analyzed 30 days post-infection. All immunized, infected mice displayed significant levels of IFN-γ production: BCGsc (3964 ± 624 pg/mL), BCGin (4760 ± 488 pg/mL), DNA-HSP65 (4583 ± 394 pg/mL), BCGin/DNA (6618 ± 806 pg/mL) and BCGsc/DNA (6067 ± 902 pg/mL) in relation to infected mice (2103 ± 488 pg/mL) (Fig. 3A). However, the BCGin/DNA and BCGsc/DNA immunized, infected mice presented higher levels of IFN-γ than other groups analyzed (Fig. 3A). Besides, BCGin/DNA group presented significant production of IFN-γ compared with BCGsc, BCGin and DNA-HSP65 (Fig. 3A) groups. We also observed that all immunized, infected mice produced significant levels of IL-12: BCGsc (3507 ± 541 pg/mL), BCGin (3267 ± 334 pg/mL), DNA-HSP65 (3120 ± 271 pg/mL), BCGin/DNA (5187 ± 855 pg/mL) and BCGsc/DNA (3158 ± 569 pg/mL) in relation to infected mice (Fig. 3B). The levels of IL-12 produced in the lung homogenates from BCGin/DNA immunized, infected mice were significantly higher than those produced by BCGin, BCGsc and DNA-HSP65-immunized, infected mice (Fig. 3B). Analysis of IL-10 revealed that mice from BCGsc (643 ± 121 pg/mL), BCGin (696 ± 63 pg/mL) and DNA-HSP65 (720 ± 82 pg/mL) groups produced lower levels of IL-10 in the lungs in comparison with the levels produced in the homogenates of mice immunized with the "prime-boost" schedule: BCGin/DNA (1097 ± 101 pg/mL) and BCGsc/DNA (1036 ± 89 pg/mL) (Fig. 3C). In addition, the levels of IL-10 produced by BCGin/DNA group were significantly different from those of BCGsc, BCGin and DNA-HSP65 groups (Fig. 3C). We also observed significant production of TNF-α in the lungs of BCGsc (506 ± 145 pg/mL), BCGin (548 ± 111 pg/mL), DNA-HSP65 (461 ± 85 pg/mL), BCGin/DNA (600 ± 51 pg/mL) and BCGsc/DNA (510 ± 76 pg/mL) groups. TNF-α secretion was very similar among the different groups of immunized mice (Fig. 3D). For BCGin/DNA immunized, infected mice and infected group, we established an inverse correlation between the levels of IFN-γ in the lungs and the numbers of CFU (Fig. 3E). Surprisingly, we observed a positive correlation between the levels of IFN-γ and IL-10 produced in the lung homogenates. This correlation was higher in the lungs of BCGin/DNA mice and lower in those of infected mice (Fig. 3F). Interestingly, the levels of IFN-γ were four times higher than the levels of IL-10.

When we analyzed the cytokine production in all immunized mice that were challenged 60 days after the last immunization, we verified that only BCGin and BCGin/DNA immunized, infected mice produced significant levels of IFN-γ in relation to infected group on days 30 and 70 post-infection (Table 2). Notably, the levels of IFN-γ produced by these two groups were significantly different from those of BCGsc immunized, infected mice after 30 and 70 days of infection and only BCGin/DNA presented significant IFN-γ production in relation to DNA-HSP65 group (Table 2). Similar results were observed when we analyzed the production of IL-12 (Table 2). On the other hand, we verified that all immunized, infected mice displayed significant production of IL-10 compared with infected group on days 30 and 70 post-infection (Table 2). Differently, only BCGin/DNA immunized, infected mice presented significant IL-10 production in relation to BCGsc, BCGin and DNA-HSP65 groups, on days 30 and 70 after infection (Table 2).

**Table 2:** Cytokine production in lung homogenates after 30 and 70 days of challenge

**Cytokines**	**time after challenge**	**non-infected mice**	**infected mice**	**immunized with (below) and infected with H37Rv**			
				**BCGsc**	**BCGin**	**DNA-HSP65**	**BCGin/DNA**
				
**IFN-γ (pg/mL)**	30 days	692 ± 155	1069 ± 170	890 ± 87	1436 ± 185^◆, ●^	1332 ± 138	2343 ± 397^◆, *^
	70 days	634 ± 110	795 ± 98	866 ± 87	1473 ± 120^◆, ●^	906 ± 64	1753 ± 190^◆, *^
**IL-12 (pg/mL)**	30 days	606 ± 111	1281 ± 146	1378 ± 213	1990 ± 310^◆, ●^	1624 ± 254	3476 ± 375^◆, *^
	70 days	447 ± 131	926 ± 56	1090 ± 153	1562 ± 180^◆, ●^	1192 ± 186	2709 ± 322^◆, *^
**IL-10 (pg/mL)**	30 days	42 ± 9	104 ± 18	430 ± 23^◆^	625 ± 79^◆, ●^	558 ± 25^◆^	684 ± 104^◆, *^
	70 days	30 ± 9	84 ± 4	391 ± 58^◆^	407 ± 33^◆^	334 ± 14^◆^	572 ± 46^◆, *^
**TNF-α (pg/mL)**	30 days	178 ± 41	328 ± 25	505 ± 39^◆^	506 ± 58^◆^	470 ± 45^◆^	662 ± 65^◆, *^
	70 days	181 ± 43	388 ± 25	530 ± 22^◆^	574 ± 44^◆^	523 ± 58^◆^	710 ± 52^◆, *^

Results were determined by ELISA in lung homogenates 30 or 70 days after infection. Mice were immunized as described in table I and 60 days after the last immunization they were challenged with *M. tuberculosis*. **30 days: **(IFN-gamma): ◆ BCGin and BCGin/DNA vs Infected mice. ● BCGin vs BCGsc. * BCGin/DNA vs BCGsc, BCGin and DNA-HSP65/(IL-12): ◆ BCGin and BCGin/DNA vs Infected mice. ● BCGin vs BCGsc. * BCGin/DNA vs BCGsc, BCGin and DNA-HSP65/(IL-10): ◆ All immunized-infected mice vs Infected mice. ● BCGin vs BCGsc. * BCGin/DNA vs BCGsc, BCGin and DNA-HSP65/(TNF-alpha): ◆ All immunized-infected mice vc Infected mice. * BCGin/DNA vs BCGsc, BCGin and DNA-HSP65. **70 days: **(IFN-gamma): ◆ BCGin and BCGin/DNA vs Infected mice. ● BCGin vs BCGsc. * BCGin/DNA vs BCGin and DNA-HSP65./(IL-12): ◆ BCGin and BCGin/DNA vs Infected mice. ● BCGin vs BCGsc. * BCGin/DNA vs BCGsc, BCGin and DNA-HSP65/(IL-10): ◆ All immunized-infect mice vs Infected mice. * BCGin/DNA vs BCGsc, BCGin and DNA-HSP65/(TNF-alpha): ◆ All immunized-infected mice vs Infected mice. * BCGin/DNA vs BCGsc, BCGin and DNA-HSP65.

### BCGin/DNA induces up-regulation of CD44^hi^/CD62L^lo ^expression in pulmonary T lymphocytes

**Figure 4:**
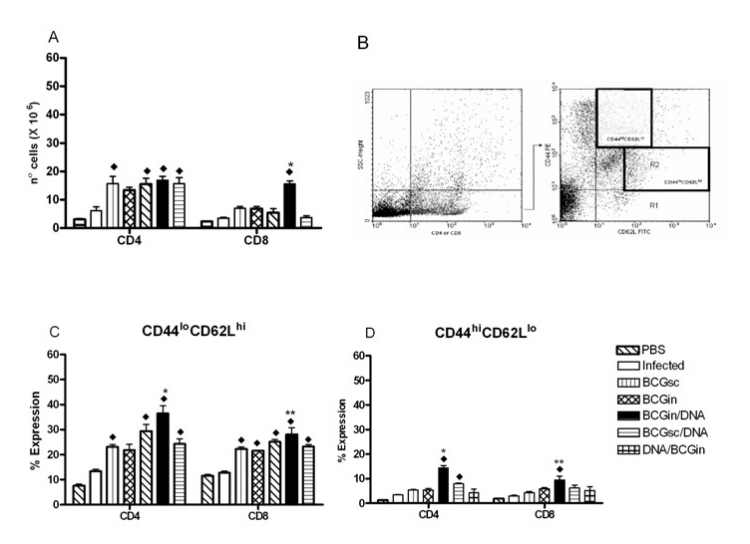
CD4^+^/CD8^+ ^cell numbers and expression of CD44^hi^CD62L^lo ^or CD44^lo^/CD62L^hi ^in the lungs of mice from the various experimental groups. (A) CD4^+^/CD8^+ ^cell numbers in the lungs. (B) Representative cell gating data showing the separation of effector cell populations. (C) CD44^lo^/CD62L^hi ^expression on CD4^+ ^and CD8^+ ^lung cells. (D) CD44^hi^/CD62L^lo ^expression on CD4^+ ^and CD8^+ ^lung cells. Groups of 7 mice were immunized according table I and 15 days after the last immunization, they were challenged with H37Rv. After 30 days of infection, the lungs were removed and analyzed by flow cytometry for cell population and expression of cell surface molecules. (A) ◆ All immunized-infected mice vs Infected mice. * BCGin/DNA vs BCGsc, BCGin and DNA-HSP65. (C) ◆ All immunized-infected mice vs Infected mice. * BCGin/DNA vs BCGin, BCGsc and DNA-HSP65.** BCGin/DNA vs BCGin. (D) ◆ All immunized-infected mice vs Infected mice. * BCGin/DNA vs BCGsc, BCGin, DNA-HSP65 and BCGsc/DNA. ** BCGin/DNA vs BCGsc. Bars represent the mean ± standard deviation. p < 0.05 was considered significant. Data are representative of two experiments.

Two lymphocyte populations were evaluated regarding the expression of CD44 and CD62L. With this in mind, we intended to study lung activated/memory cells. Firstly, we verified that all immunized, infected mice, with the exception of BCGin group, exhibited a significant CD4^+ ^cells influx into lungs when compared with infected mice (Fig. 4A). However, we did not observe significant differences among the groups (Fig. 4A). On the other hand, only BCGin/DNA-immunized, infected mice presented a significant influx of CD8^+ ^cells not only when compared with infected mice but also when compared with BCGsc, BCGin and DNA-HSP65 groups (Fig. 4A). When we analyzed the expression of CD44^lo ^and CD62L^hi ^molecules on CD4^+ ^lymphocytes, we found variations in the percentage of expression among all immunized-infected groups. In comparison with the infected group, all immunized, infected mice presented higher expression of these molecules on CD4^+ ^cells (Fig. 4C). Moreover, BCGin/DNA group presented significant expression in relation to other groups (Fig. 4C). We also observed that the expression of CD44^lo ^and CD62L^hi ^molecules on CD8^+^lymphocytes was similar among all immunized, infected mice (Fig. 4C). We verified that BCGin/DNA and BCGsc/DNA groups presented significant expression of CD44^hi ^and CD62L^lo ^on CD4^+ ^cells when compared with infected group (Fig. 4D). Moreover, BCGin/DNA group also presented significant expression of CD44^hi ^and CD62L^lo ^on CD4^+ ^cells in relation to BCGsc, BCGin, DNA-HSP65 and BCGsc/DNA groups (Fig. 4D). On top of that, only BCGin/DNA group presented significant expression of CD44^hi^CD62L^lo ^molecules on CD8^+ ^cells in relation to infected mice (Fig. 4D). We also evaluated these cell populations 30 and 70 days after mycobacterial infection. Thirty days after infection we verified a significant CD4^+ ^cells influx into lungs in all immunized, infected mice when compared with non-immunized infected mice and non-infected mice (Table 3). In contrast, only BCGin/DNA group presented a significant influx of CD4^+ ^cells after 70 days of infection (Table 3). Analysis of CD8^+ ^cells influx into lungs revealed that all immunized, infected mice presented a significant influx of CD8^+ ^cells in relation to infected mice 30 days post-infection (Table 3). When we analyzed the percentage of CD4^+ ^or CD8^+ ^cells expressing CD44^hi^CD62L^lo ^30 or 70 days post-infection, we verified that only BCGin/DNA group presented significant expression of CD44^hi^CD62L^lo^molecules on CD4^+ ^cells in relation to BCGsc, DNA-HSP65 and infected mice 70 days post-infection (Table 3). Besides, BCGin/DNA immunized, infected mice also presented significant expression of CD44^hi^CD62L^lo ^on CD8^+ ^cells when compared with BCGin group 30 days post-infection (Table 3). On the other hand, 70 days after infection BCGin/DNA group exhibited a significant expression of these molecules on CD8^+ ^cells in relation to other groups: infected mice, BCGsc, BCGin and DNA-HSP65 immunized, infected mice (Table 3). The expression of CD44^lo^and CD62L^hi ^was also analyzed in the same cell population. Thirty days after infection, we observed that all immunized mice presented significant percentage of CD4^+ ^cells expressing CD44^lo^CD62L^hi ^molecules in relation to infected mice (Table 3). This expression was significant when we compared the BCGin/DNA group with BCGsc, BCGin and DNA-HSP65 immunized, infected mice (Table 3). Equivalent analyses performed 70 days post-infection revealed that only BCGin and BCGin/DNA groups presented significant expression of CD44^lo^CD62L^hi ^on CD4^+ ^cells compared with infected group (Table 3). We also observed that BCGin and BCGin/DNA groups presented significant expression of CD44^lo^CD62L^hi ^on CD8^+ ^cells in relation to infected mice 30 days after infection (Table 3). Nevertheless, only BCGin/DNA group presented significant expression of CD44^lo^CD62L^hi ^molecules on CD8^+ ^cells after 70 days of infection.

**Table 3:** CD4^+^/CD8^+ ^cell numbers and expression of CD44^hi^CD62L^lo ^or CD44^lo^/CD62L^hi ^in the lungs of mice

	**time after challenge**	**non-infected mice**	**infected mice**	**immunized with (below) and infected with H37Rv**			
				**BCGsc**	**BCGin**	**DNA-HSP65**	**BCGin/DNA**
				
**CD4**^+^	30 days 70 days	2.4 ± 0,6 1 ± 0,1	5 ± 0,8 1,6 ± 0,2	9 ± 0,7^◆^ 1,7 ± 0,6	10,2 ± 0,5^◆^ 2,18 ± 0,7	10,2 ± 0,8^◆^ 1,64 ± 0,1	12,8 ± 1,5^◆, *^ 2,8 ± 0,5^◆, *^
**CD8**^+^	30 days	1,4 ± 0,2	2,5 ± 0,06	4,5 ± 0,4^◆^	5,9 ± 0,6^◆^	4,7 ± 1,5^◆^	6,2 ± 1,5^◆^
	70 days	0,6 ± 0,3	1 ± 0,3	1 ± 0,4	1,3 ± 0,3	1 ± 0,1	1,4 ± 0,2
**CD4^+^/CD44^hi^/CD62L^lo^**	30 days	6,1 ± 2	12,8 ± 2,6	10 ± 4,7	10,7 ± 2,5	11,8 ± 2	15,3 ± 4
	70 days	1,5 ± 0,5	3,6 ± 0,9	4,5 ± 1,4	6 ± 1	3,4 ± 0,9	9 ± 2,2^◆, *^
**CD8^+^/CD44^hi^/CD62L^lo^**	30 days	7,2 ± 1,4	14,6 ± 3,1	14,8 ± 6,4	12,8 ± 4	14,5 ± 1,3	20,8 ± 0,6*
	70 days	1 ± 0,3	2,5 ± 0,7	2 ± 0,8	2,2 ± 1,7	2,7 ± 0,3	5,2 ± 0,5^◆, *^
**CD4^+^CD44^lo^/CD62L^hi^**	30 days	14 ± 1,5	17 ± 1,42	19,5 ± 2,6^◆^	36,8 ± 5,4^◆, ●^	24,6 ± 2,5^◆^	35,6 ± 4,3^◆, *^
	70 days	12 ± 2,4	22,2 ± 1,27	26,2 ± 7,5	37,5 ± 5^◆, ●^	30 ± 3,8	42,4 ± 5,4^◆, *^
**CD8^+^CD44^lo^/CD62L^hi^**	30 days	15,3 ± 2	19,38 ± 1,7	21,9 ± 4,1	29 ± 2,8^◆^	18,42 ± 5,6	41,2 ± 6,5^◆, *^
	70 days	8,7 ± 2	16 ± 4,4	24,3 ± 7,1	22,6 ± 1,1^◆, ●^	27,45 ± 6,1	35,2 ± 7,3^◆, *^

Results were determined by flow cytometry. Number of CD4+ and CD8+ × 10^6 ^cells/mL. Expression of CD44^hi^/CD62L^lo ^or CD44^lo^/CD62L^hi ^by CD4+ or CD8+ cells was calculated in percentage from dot plots analysis. Mice were immunized as described in table I and 60 days after the last immunization they were challenged with *M. tuberculosis*. **30 days: **(CD4): ◆ All immunized-infected mice vs Infected mice. * BCGin/DNA vs BCGsc, BCGin and DNA-HSP65/(CD8): ◆ All immunized-infected mice vs Infected mice/(CD8^+^CD44^hi^CD62L^lo^): * BCGin/DNA vs BCGin/(CD4^+^CD44^lo^CD62L^hi^): ◆ All immunized-infected mice vs Infected mice. ● BCGin vs BCGsc. * BCGin/DNA vs BCGsc, DNA-HSP65/(CD8^+^CD44^lo^CD62L^hi^): ◆ BCGin and BCGin/DNA vs Infected mice. * BCGin/DNA vs BCGsc, BCGin and DNA-HSP65. **70 days: **(CD4): ◆ BCGin/DNA vs Infected mice. * BCGin/DNA vs BCGsc and DNA-HSP65/(CD4^+^CD44^hi^CD62L^lo^): ◆ BCGin/DNA vs Infected mice. * BCGin/DNA vs BCGsc, BCGin and DNA-HSP65/(CD8^+^CD44^hi^CD62L^lo^): ◆ BCGin/DNA vs Infected mice. * BCGin/DNA vs BCGsc, BCGin and DNA-HSP65/(CD4^+^CD44^lo^CD62L^hi^): ◆ BCGin and BCGin/DNA vs Infected mice. ● BCGin vs BCGsc. * BCGin/DNA vs BCGsc and DNA-HSP65./(CD8^+^CD44^lo^CD62L^hi^): ◆ BCGin/DNA and BCGin vs Infected mice. ● BCGin vs BCGsc. * BCGin/DNA vs BCGsc, DNA-HSP65.

### Reduction of lung injury after BCGin/DNA vaccination

**Figure 5:**
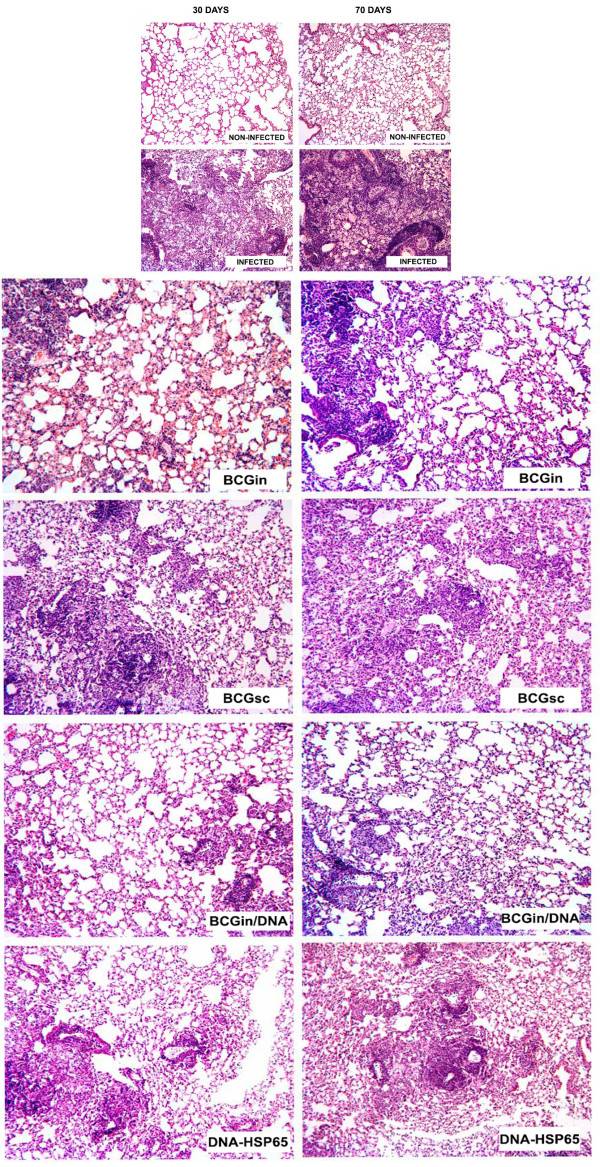
Profile of the inflammatory infiltrates in lungs of mice that were immunized, as described in table I and infected with H37Rv (10^5 ^bacilli/mouse) by intratracheal route. Thirty and 70 days after infection, the lungs were removed and the histological analysis were performed in H.E. staining. Small pictures represent the controls (infected and non-infected mice). The main figure shows all immunized, infected mice as observed in the individual description per figure. The left column represent 30 days and the right one represent 70 days after infection. Original magnification: 40×.

Histological sections of control mice are presented in 4 small pictures above the main figure (Fig. 5). Infected mice presented extensive damage in the pulmonary parenchyma, characterized by confluent granulomas. An exacerbation of pulmonary infection, with a more severe alveolar injury was observed 70 days after infection (Fig. 5, small). Lungs of non-infected mice presented a normal alveolar architecture (Fig. 5, small).

Thirty days after infection the lungs of BCGin immunized mice presented few granulomas with mild parenchyma injury. After 70 days of infection, this group was characterized by a more extensive inflammatory response (Fig. 5X). In the lungs of BCGsc mice, we observed sparse, well-defined granulomas, containing macrophages and surrounded by a few lymphocytic foci. These mice presented less parenchymal damage than did infected mice, but on day 70 after infection the granulomatous process was more intense than that observed on day 30 post-infection (Fig. 5C). In the BCGin/DNA group, the lung parenchyma presented less damage and smaller foci of mononuclear inflammatory infiltrates than in any other group. This infiltration was characterized by the presence of macrophages and few lymphocytes, as well as by rare granulomatous lesions. Similar characteristics were observed 70 days after infection (Fig. 5D). The lungs of DNA-HSP65 immunized mice presented compact granulomas with mild parenchyma damage after 30 days of infection. Conversely, on day 70 post-infection, an intense inflammatory reaction characterized by the presence of multiples granulomas and increased tecidual damage was observed (Fig. 5X). It is noteworthy that both groups receiving BCGin prime (BCGin/DNA, Fig. 5) presented less parenchymal injury than did those receiving BCGsc prime (Fig. 5X).

## Discussion

In this study, we showed that heterologous prime-boost vaccination using intranasal BCG priming/DNA-HSP65 boosting (BCGin/DNA) provided significantly greater protection than that afforded by a single subcutaneous or intranasal dose of BCG. In addition, BCGin/DNA immunization was also more efficient in controlling bacterial loads when compared with the other prime-boost schedules (data not shown) evaluated or three doses of DNA-HSP65 as a naked DNA. The DNA-HSP65 booster enhanced the immunogenicity of a single subcutaneous BCG vaccination, as evidenced by the significantly higher serum levels of anti-Hsp65 IgG2a Th1-induced antibodies, as well as by the significantly greater production of IFN-γ by antigen-specific spleen cells. The BCGin prime was also associated with better preservation of lung parenchyma. Our findings also suggest that the order of stimuli is more relevant to the modulation of immune responses after challenge than is the route of BCG administration. Despite the fact that BCGin/DNA immunization clearly induced greater protection than did BCGsc/DNA immunization, both stimulated similar levels of IFN-γ production.

Distinct prime-boost vaccination protocols have been evaluated in experimental TB models. Goonetilleke et al. reported that parenteral or intranasal BCG immunization induced comparable levels of antigen-specific CD4^+ ^responses in the spleen [11]. However, only intranasal BCG (BCGin) elicited specific T cell responses in the lungs. We demonstrated that, although parenteral and intranasal prime induced comparable IFN-γ levels at the site of the infection, the latter clearly decreased the bacterial load on the order of 3 log_10_, in relation to non-immunized, infected mice, and did not provoke lung injury when the challenge was performed 15 days after immunization schedules. A difference of 1,2 LOG_10 _between the same groups was verified when the challenge was performed 60 days after vaccination. In a similar prime-boost strategy, Mollenkopf et al. showed that a DNA vaccine improved the efficacy of intravenous BCG prime [15]. Likewise, our results reinforce the hypothesis that a DNA booster can increase BCG immunogenicity. Notably, in our study, a single booster with DNA-HSP65 conferred considerable protection. Two main aspects merit emphasis. The first is that intranasal route employed in our study is less invasive, primes the lymphoid tissues (in the nasal and bronchial mucosa) and is easily applied in humans [16]. The second is that, in addition to increasing BCG-related protection, prime-boost immunization also makes it possible to optimize DNA-HSP65 immunization. In a classical protocol of DNA vaccination, we employed a schedule of 3 or 4 doses at 15-day intervals. In previous studies, the protective efficacy of DNA vaccine has been demonstrated [2,4,5,17]. Nevertheless, other authors have found that administration of a DNA vaccine provokes a pronounced, disorganized granulomatous response that leads to consolidation of lung tissues, and that there was no evident protection, whether the vaccine was used prophylactically or therapeutically [18]. In an attempt to increase the protective effect and to minimize possible side effects of DNA-HSP65 vaccine, we included the BCGin/DNA prime-boost strategy in our study. This strategy exceeded our expectations when the perspectives described above were attended by a single DNA administration.

To understand the possible mechanisms involved in the up-modulation of the immune response, we sought and found a correlation between IFN-γ and IL-10 levels, as well as between IFN-γ levels and CFU numbers. Measuring IFN-γ production by antigen-specific T cells provides the best available immunological correlate of protection against TB [19]. Although this immunological parameter of protection is currently in question [20], the results described here show that levels of "ex vivo" IFN-γ are closely associated with protection. Surprisingly, in the lungs of sham-immunized, infected mice and BCGin/DNA immunized mice, we found a positive correlation between IFN-γ and IL-10 levels after challenge and, as expected, a negative correlation between IFN-γ levels and CFU counts. In the lungs of BCGin/DNA mice, IFN-γ levels were approximately four times higher than those of IL-10, although IL-10 production was higher than in the lungs of sham-immunized, infected mice. There is little consensus in the literature regarding the role of IL-10 in mycobacterial infection. Absence of IL-10 in the early phase of infection favors increased resistance to mycobacteria [21]. However, in IL-10 transgenic murine model, the presence of excess IL-10 did not inhibit the T cell response to mycobacteria infection. Thus, IL-10, which was initially found to be an inhibitor of IFN-γ secretion, had little effect on IFN-γ production in this experimental model. In addition, the IL-10 secreted from activated T cells appears to have little influence on the overall patterns of cytokine secretion in response to mycobacterial infection [22]. In a more recent study, Jung, 2003 demonstrated that there was no difference between wild-type and IL-10 knockout mice in their ability to deal with mycobacterial infection [23]. It seems reasonable to assume that the IFN-γ-mediated protection observed in our study was associated with the decreased bacterial load and, consequently, control of the infection, whereas the IL-10-mediated protection was, to a great extent, due to an anti-inflammatory effect related to protection against tissue damage. Since the bacterial clearance is followed by tissue repair, the two events are not mutually exclusive. However, the anti-inflammatory effect cannot be exclusively attributed to IL-10. The BCGin and BCGsc mice, despite presenting IL-10 levels that were statistically lower than those detected in mice in the other prime-boost immunized groups, also presented less parenchymal damage than did the sham-immunized mice. In this context, it is possible that an effector function is performed by soluble mediators, such as transforming growth factor-β, or by cell-cell contact mediated by regulatory T cells, although this has yet to be investigated. It is also of note that TNF-α levels were comparable among the various experimental groups. Since one of the strongest correlates of TNF-α-mediated protection is its role in granuloma formation [24], we expected to find differences among the groups. Although the numbers of CD8^+ ^cells in the lungs were significantly higher in BCGin/DNA immunized mice, TNF-α levels were comparable among the groups, despite variations in cell number and cell constitution. These data should motivate a search for differential cytokines, chemokines and adhesion receptors that might prove to be markers of disease progression.

We also analyzed the effector/memory phenotype of T lymphocytes in the lungs. It is well known that effector cells express CD62L^lo ^and CD44^hi ^and are characteristically short-lived [25]. We have previously shown that expression of CD44^hi ^on CD4^+ ^and CD8^+ ^cells is related to protection against TB [2,6]. However, Kipnis et al. recently reported that the transference of spleen cells expressing a resting/naïve phenotype (CD62L^hi^/CD44^lo^) but not effector cells (CD62L^lo^/CD44^hi^) protected the recipients after challenge with *M. tuberculosis *[26]. The authors suggested that the emergence of T cell memory from the naive subset induces IFN-γ-mediated protection. We found an increase of CD4^+ ^and CD8^+ ^populations that express CD44^hi^CD62L^lo ^in the lungs of BCGin/DNA, however we did not find striking differences in the CD44^lo^CD62L^hi ^populations among the distinct groups of immunized, infected mice.

In conclusion, we found that a DNA-HSP65 booster increased BCG-mediated protection and that the order of stimulation was a relevant correlate for this protection. This makes the BCGin/DNA strategy attractive because it does not preclude childhood BCG vaccination. Increased protective efficacy induced by the DNA-HSP65 booster appeared to be attributable to increased numbers of CD4^+ ^and CD8^+ ^cells expressing the effector phenotype CD62L^lo^/CD44^hi^, as well as to higher IFN-γ, IL-10 and IL-12 levels.

## Abbreviations

BCG – Bacillus Calmette-Guerin

BCGin – intranasal administration of the BCG

BCGsc – subcutaneous administration of the BCG

CFU – colony forming unit(s)

DNA-HSP65 – DNA vaccine encoding the *M. leprae *65-kDa heat shock protein

IFN-γ interferon-gamma

IL – interleukin

MVA – modified vaccinia virus Ankara

PBS – phosphate buffered saline

rhsp65 – recombinant 65-kDa heat shock protein

TB – tuberculosis

TNF-α tumor necrosis factor-alpha

Con-A concanavalin A

## Authors' contributions

Thirteen researchers participated in this study. EDCG, VLDB and CLS are the principal investigators in this study. EGS participated in the histological analysis. Experiments involving mice were done by DMF, ITB and APSM in the laboratory of CLS and the Company Farmacore Biotecnologia Ltda, who also shared their expertise in the DNA vaccine. The majority of the research was done in the laboratory of CLS who coordinated, together with VLDB, the project and provided critical input and assistance.
